# Beyond open access: open discourse, the next great equalizer

**DOI:** 10.1186/1742-4690-3-55

**Published:** 2006-08-30

**Authors:** Andrew I Dayton

**Affiliations:** 1 Laboratory of Molecular Virology, Division of Emerging and Transfusion Transmitted Diseases, Center for Biologics Evaluation and Research, FDA, HFM 315 FDA/CBER, 1401 Rockville Pike, Rockville, MD, 20852-1448, USA

## Abstract

The internet is expanding the realm of scientific publishing to include free and open public debate of published papers. Journals are beginning to support web posting of comments on their published articles and independent organizations are providing centralized web sites for posting comments about any published article. The trend promises to give one and all access to read and contribute to cutting edge scientific criticism and debate.

If you are reading this you are benefiting from the Open Access movement in scientific publishing. Open Access reduces the great divide between the haves and have-nots of the scientific world, allowing anyone, anywhere on the planet with internet access to read with full text and graphics the latest scientific reports, unfettered by prohibitive subscription fees or lack of affiliation with a major institution to pay for them. That the process directly delivers to the public a product paid for by their taxes can only be considered a just and additional benefit. But access to cutting edge knowledge is not the only divide between the haves and the have-nots. Even Open Access leaves a vast inequality in scientific discourse. If you can't afford to attend the latest scientific meetings (say, for instance, you work for the US government) or are not a member of a prestigious institution, you can be frozen out of cutting edge scientific discussions. You can neither query the major players nor contribute to the debates, unless your prestige or the media value of the subject matter is such to garner you a published letter to the editor. You can't even witness the debates until they are published in review articles, by which time they are mostly over.

How often have you asked yourself how a certain study was published unchallenged, without the results of a key control? How often have you wondered whether a paper's authors performed a specific procedure correctly? How often have you had the opportunity to question authors about previously published or opposing results they failed to cite, or discuss the difficulties of reproducing certain results? How often have you had the opportunity to command a discussion of an internal contradiction the referees seemed to have missed? The haves of science, who benefit from the *status quo *they shepherd, have seldom felt the need to redress such grievances. The have nots have basically been stuck with their lot – until now.

Enter *JournalReview.org *[1], a website forum for open peer review and discussion/criticism of medical literature. Essentially an online journal club with free membership, *JournalReview.org *provides a venue which will improve communication among physicians and scientists and foster comment and criticism about published scientific research. The goal is a better understanding and interpretation of medical literature. *JournalReview.org *has no political or commercial affiliations and was created solely by the work of two physicians (Jeffrey Ellis, Adam Penstein), one medical student (Lori Ellis) and one computer programmer (Aryeh Goldsmith).

How does it work? Simple: the site lists journals available for discussion. Under the "Basic Science" heading are a number of general purpose and specialty journals typically of interest to retrovirologists, including Nature, Cell, Science and this journal, among others. In future it is hoped the list of journals will grow in concert with interest in Open Discourse. By navigating to the journal and article of interest within a relevant discipline, anyone can initiate, read, or add to a discussion as they see fit. Currently journals are listed according to discipline. Corresponding authors are notified of comments submitted to the discussions of their articles to facilitate timely responses.

Central to the process is anonymity, which is the default option if posters do not self identify in the post. Though this can lead to abuses (and what human endeavor can not?) it allows the unempowered of science to challenge the empowered. But it would do a great disservice to Open Discourse to promote it as supporting would be Davids against reigning Goliaths. Just as Open Access distributes primary knowledge, Open Discourse distributes debate. It enables under privileged students, and even citizens, in the third world to witness the unfolding of science in real time. It can forewarn them of the drawbacks to seemingly convincing, but flawed work, saving them time and possibly resources. At another level, Open Discourse can raise the quality of journal clubs by accelerating progress through previously debated issues, and allowing participants to move on more quickly to the next levels of discussion. It can accelerate mastery of a field by those newly moving into it. And though the process may be painful for authors, it will give them opportunity to publicly defend their work and enlarge the discussion of it post publication. In short, everybody wins. Examples of discussions underway can be found by navigating to the Dermatology section of *JournalReview.org *[2] and choosing from the list of "Recent Reviews." If this commentary is successful, I hope you will find a similar list in the Basic Science section [3].

*JournalReview.org *is not the sole source for Open Discourse. *Retrovirology *[4] and other *BioMed Central *journals [5] already provide a specific tool for all interested participants to submit comments (without anonymity, though) about a published work using the "Post a Comment" function, as illustrated in the accompanying figure (Fig. 1). A site similar to *JournalReview.org*, *BioWizard *[6], hosts commentaries, but only on articles reached by searching through *PubMed *[7], and requires posters to at least identify their institutions and cities. *PLoS ONE *[8] plans to offer commentary on its publications, once it is launched. Even the dowager empress of biological journals, *Cell *[9], has ventured a cautious toe to the tide, inviting public commentary on selectively "featured" articles. The concept, it seems, is coming of age.

**Figure 1:**
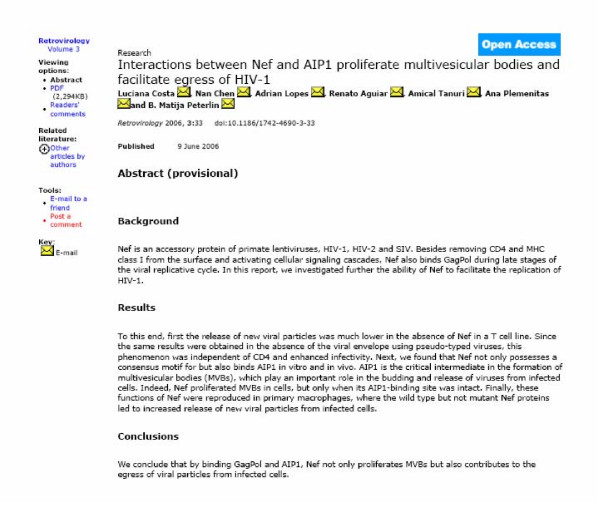
*Retrovirology *and other *BioMed Central *publications provide a "Post a comment" button to append commentary to their published articles, such as the one displayed [10].

So let us invite ourselves to commit to Open Discourse. Let us set the tone and establish the precedent of enlightened debate that is public spirited, as well as public. Let us refrain from contributing the inconsequential, the self serving and the counterproductive. And above all, let us remember that discourse need not be discourteous. I encourage all of us to not only participate in this movement, but to promote it. Tell a friend. Tell a mentor. Tell a protégé. Start submitting comments. In the end the value we receive will be the value we give. And the value to the world will be greater still.

*The author has no affiliation with JournalReview.org, Biowizard.com, PLoS ONE, nor any competing interests. Opinions expressed in this publication reflect the professional views of the author and should not be viewed as official policy of the US Food and Drug Administration or the Government of the United States. Additionally, the opinions expressed do not necessarily represent those of Retrovirology or its editorial board*.

## References

[B1] http://www.journalreview.org.

[B2] http://www.journalreview.org/spage.php?specialty_id=5&sdesc=Dermatology.

[B3] http://journalreview.org/spage.php?specialty_id=28&sdesc=Basic+Science.

[B4] http://retrovirology.com.

[B5] http://www.biomedcentral.com.

[B6] http://www.biowizard.com.

[B7] http://www.pubmed.gov.

[B8] http://www.plosone.org.

[B9] http://www.cell.com.

[B10] Costa L, Chen N, Lopes A, Aguiar R, Tanuri A, Plemenitas A, Peterlin BM (2006). Interactions between Nef and AIP1 proliferate multivesicular bodies and facilitate egress of HIV-1. Retrovirology.

